# Accurate Prediction of Transposon-Derived piRNAs by Integrating Various Sequential and Physicochemical Features

**DOI:** 10.1371/journal.pone.0153268

**Published:** 2016-04-13

**Authors:** Longqiang Luo, Dingfang Li, Wen Zhang, Shikui Tu, Xiaopeng Zhu, Gang Tian

**Affiliations:** 1 School of Mathematics and Statistics, Wuhan University, Wuhan, 430072, China; 2 School of Computer, Wuhan University, Wuhan, 430072, China; 3 Research Institute of Shenzhen, Wuhan University, Shenzhen, 518057, China; 4 Program in Bioinformatics and Integrative Biology, University of Massachusetts Medical School, 368 Plantation Street, Worcester, Massachusetts, 01605, United States of America; Harbin Institute of Technology Shenzhen Graduate School, CHINA

## Abstract

**Background:**

Piwi-interacting RNA (piRNA) is the largest class of small non-coding RNA molecules. The transposon-derived piRNA prediction can enrich the research contents of small ncRNAs as well as help to further understand generation mechanism of gamete.

**Methods:**

In this paper, we attempt to differentiate transposon-derived piRNAs from non-piRNAs based on their sequential and physicochemical features by using machine learning methods. We explore six sequence-derived features, i.e. spectrum profile, mismatch profile, subsequence profile, position-specific scoring matrix, pseudo dinucleotide composition and local structure-sequence triplet elements, and systematically evaluate their performances for transposon-derived piRNA prediction. Finally, we consider two approaches: direct combination and ensemble learning to integrate useful features and achieve high-accuracy prediction models.

**Results:**

We construct three datasets, covering three species: *Human*, *Mouse* and *Drosophila*, and evaluate the performances of prediction models by 10-fold cross validation. In the computational experiments, direct combination models achieve AUC of 0.917, 0.922 and 0.992 on *Human*, *Mouse* and *Drosophila*, respectively; ensemble learning models achieve AUC of 0.922, 0.926 and 0.994 on the three datasets.

**Conclusions:**

Compared with other state-of-the-art methods, our methods can lead to better performances. In conclusion, the proposed methods are promising for the transposon-derived piRNA prediction. The source codes and datasets are available in S1 File.

## 1. Introduction

Non-coding RNAs (ncRNAs) are important functional RNA molecules, which are not translated into proteins [1, 2]. Non-coding RNAs are classified as long ncRNAs and short ncRNAs, roughly by their length. Long ncRNAs are usually longer than 200 nucleotides [3, 4]. Among short ncRNAs, those having 20~32 nt in length are defined as small ncRNAs, such as microRNAs (miRNAs) and piwi-interacting RNAs (piRNAs) [5]. piRNA is a distinct class of small ncRNAs mainly expressed in germline cells, and its length is slightly longer than miRNA, about 26~32 nt in general [6–8]. Compared with miRNA, piRNA lacks conservative secondary structure motifs, and the presence of a 5’ uridine is common in both vertebrates and invertebrates [5, 9, 10].

piRNA plays an important role in the transposon silencing, and involves the germ cell formation, germline stem cell maintenance, spermatogenesis and oogenesis [11–15]. About nearly one-third of the fruit fly and one-half of human genomes are transposon elements. These transposons move within the genome and induce insertions, deletions, and mutations, which may cause the genome instability. piRNA pathway is an important genome defense mechanisms to maintain genome integrity. Loaded into PIWI proteins, piRNAs serve as a guide to target the transposon transcripts by sequence complementarity with mismatches, and then the transposon transcripts will be cleaved and degraded, producing secondary piRNAs, which is called ping-pong cycle in fruit fly [13–17]. Therefore, predicting transposon-derived piRNAs provides biological significance and insights into the piRNA pathway.

The wet method combines immunoprecipitation and deep sequencing to recognize piRNAs [18], but the diversity and non-conservation of piRNAs make the work complicated [5, 9, 10]. To the best of our knowledge, several computational methods have been proposed for piRNA prediction. Betel *et al*. developed the position-specific usage method to identify piRNAs [19]. Zhang *et al*. utilized a *k*-mer feature, and adopted support vector machine (SVM) to build the classifier (named piRNApredictor) for piRNA prediction [20]. Wang *et al*. proposed a method named Piano to predict piRNAs. They utilized the piRNA-transposon interaction information to extract feature vector and used SVM to build prediction models [21].

Following the pioneering works: Betel’s method [19], piRNApredictor [20] and Piano [21], we attempt to differentiate transposon-derived piRNAs from non-piRNAs based on their sequential and physicochemical features. Features are critical for the construction of prediction models. Since piRNA sequences have varied lengths, we explore six useful features: spectrum profile [22–25], mismatch profile [25, 26], subsequence profile [25, 27], position-specific scoring matrix [28–30], pseudo dinucleotide composition [23, 24], local structure-sequence triplet elements [21, 31], which can transform piRNA sequences into fixed-length feature vectors. Then, we systematically evaluate these sequence features, and discuss how to integrate these features for high-accuracy performances. In this paper, we consider two feature combination approaches. The first one, named direct combination, is to merge different feature vectors. Another one is ensemble learning, which uses the weighted average scores of individual feature-based predictors. According to the experiments, both direct combination and ensemble learning achieve AUC of >90% and accuracy of >80% on three datasets (*Human*, *Mouse* and *Drosophila*).

## 2. Materials and Methods

### 2.1. Datasets

In this paper, we construct three datasets: *Human*, *Mouse* and *Drosophila*, and the data compiling procedures are described as follows.

For *Human* dataset, we download 32,152 *Human* piRNAs from the NONCODE version 3.0 [32], 5,520,017 *Human* repeats and all *Human* chromosomes from the UCSC Genome Browser (hg38) [33]. Then, we extract *Human* transposons from the *Human* repeats. After aligning piRNAs to *Human* transposons with SeqMap (three mismatches at most) [34], 7,405 non-redundant *Human* piRNAs are obtained as positive samples. We also download 59,003 *Human* non-piRNA ncRNAs from the NONCODE version 3.0 [32], and remove non-piRNA ncRNAs whose lengths are shorter than the minimum length of positive samples. Then, we randomly cut out short sequences as the candidate pseudo piRNAs from each non-piRNA ncRNA. After aligning them to *Human* transposons, 68,654 non-redundant candidate pseudo piRNAs are obtained.

As far as we know, for the bioinformatics molecular identification problems, the negative samples are always far more than positive ones. Lots of computational works have discussed how to select negative samples to compile datasets [35, 36]. Since latest transposon-derived piRNA prediction method (Piano) adopt this strategy which select almost the same number of negative samples as positive samples [21], and we follow it in order to make fair comparison. Therefore, we randomly select pseudo piRNAs from 68,654 non-redundant candidate pseudo piRNAs to simulate the number and length distribution of positive samples. Finally, 7,405 non-redundant pseudo piRNAs are generated as the negative samples.

Further, in the same way, we download 75,814 *Mouse* piRNAs from the NONCODE version 3.0 [32] and 12,903 *Drosophila* piRNAs (GSE9138) from the NCBI Gene Expression Omnibus [18]. Then, we obtain 3,660,356 *Mouse* (mm10) and 37,326 *Drosophila* (dm6) transposons from the UCSC Genome Browser [33]. After aligning these piRNAs to their relevant transposons, 13,998 *Mouse* and 9,214 *Drosophila* piRNAs are obtained. The construction of pseudo piRNAs of *Mouse* and *Drosophila* datasets is similar to the construction of *Human* negative samples.

Three datasets are summarized in Table 1.

**Table 1:** Datasets for piRNA prediction.

*Dataset*	*Positive Samples*	*Negative Samples*
***Human***	***7*,*405***	***7*,*405***
***Mouse***	***13*,*998***	***13*,*998***
***Drosophila***	***9*,*214***	***9*,*214***

### 2.2. Features

For prediction, we should explore informative features that can characterize piRNAs and convert flexible-length piRNA sequences into fixed-length feature vectors. Here, we consider six potential features: spectrum profile [22–25], mismatch profile [25, 26], subsequence profile [25, 27], position-specific scoring matrix [28–30], pseudo dinucleotide composition [23, 24], local structure-sequence triplet elements [21, 31]. Among six features, the spectrum profile and the local structure-sequence triplet elements were ever adopted for piRNA prediction by Zhang *et al*. [20] and Wang *et al*. [21], respectively. The mismatch profile, subsequence profile, position-specific scoring matrix and pseudo dinucleotide composition are widely used for biological sequence analysis [23–30], but are never used in the piRNA prediction. These sequence-derived features are briefly introduced as follows.

#### 2.2.1. Spectrum profile

Spectrum profile is to count the repeated patterns of sequences, and its success has been proved by numerous bioinformatics applications [22–25]. piRNA sequences consist of four types of nucleotides *A*, *C*, *G* and *T*. In the sequence analysis, the repeated patterns are denoted as *k*-mers (*k* is a parameter, *k* ≥ 1), namely *k*-length contiguous strings. There are totally 4^*k*^ *k*-mers for a given *k*. For example, we have 64 types of 3-mers: *AAA*, *AAC*, …, *TTT*.

Given a nucleotide sequence *x*, the spectrum profile of sequence *x* is defined as:

$$f_{k}^{spe}(x)=(c_{1},c_{2},\ldots ,c_{4^{k}})$$

where *c*_*i*_ represents the occurrences of different *k*-mers in *x*, *i* = 1,2,…,4^*k*^.

#### 2.2.2. Mismatch profile

Mismatch profile also calculates the occurrences of *k*-mers, but allows max *m* inexact matching (*m* < *k*) [25, 26]. For 3-mer “*AAC*” and max one mismatch, we should consider the substrings: *AAA*, *AAC*, *AAG*, *AAT*, …, *CAC*, *GAC*, *TAC* in the sequences, and take them as the occurrences of “*AAC*”. The mismatch profile of sequence *x* is defined as:

$$f_{k,m}^{mis}(x)=(\sum_{j=0}^{m}c_{1,j},\sum_{j=0}^{m}c_{2,j},\ldots ,\sum_{j=0}^{m}c_{4^{k},j})$$

where *c*_*i*,*j*_ represents the occurrences of *i-*th *k*-mer type in *x*, having just *j* mismatches, *i* = 1,2,…,4^*k*^; *j* = 0,1,…,*m*.

#### 2.2.3. Subsequence profile

Subsequence profile allows non-contiguous matching [25, 26]. For example, we want to search the 3-mer “*AAC*” in the sequence “*AACTACG*”. By exact and non-contiguous matching, we can obtain *AAC*, *AA—C*, *A—AC*, *A—AC* (“-” means the gap in non-contiguous matching). *AAC* is the exact form of “*AAC*”, and *AA—C*, *A—AC*, *A—AC* are non-contiguous forms of “*AAC*”. The occurrences of non-contiguous forms are penalized with their length *l* and the factor *δ* (0 ≤ *δ* ≤ 1), defined as *δ*^*l*^. Therefore, the occurrence of “*AAC*” in above example is 1 + 2*δ*^6^ + *δ*^5^. The subsequence profile of sequence *x* is defined as:

$$f_{k,\delta }^{sub}(x)=(c_{1,\delta },c_{2,\delta },\ldots ,c_{4^{k},\delta })$$

where

$$c_{i,\delta }=\sum_{k-\text{mer}\;\alpha_{i}\;\text{in}\;x}\delta^{l(\alpha_{i})},\;i=1,2,\ldots ,4^{k}$$

and *l*(*α*_*i*_) is given as:

$$l(\alpha_{i})\left\{ \begin{array}{l} 0,\;\alpha_{i}\;\text{is exact matching}\;;\\ |\alpha_{i}|,\;\alpha_{i}\;\text{is non-contiguous matching}. \end{array}\right.$$

where |*α*_*i*_| represents the length of *α*_*i*_, *i* = 1,2,…,4^*k*^.

#### 2.2.4. Position-specific scoring matrix

Position-Specific Scoring Matrix (PSSM) is popular for representing patterns in biological sequences [28–30]. PSSM is usually generated from the fixed-length sequences. Since piRNA sequences have varied lengths, we have to process sequences to meet requirements. Here, we set the fixed length of sequences as *d*. We truncate the first *d* nucleotides of long sequences which lengths are more than *d*; the empty symbols “*E*” are added at end of short sequences. Therefore, all flexible sequences are transformed into fixed-length sequences, and PSSM can be calculated on training dataset.

In the training and testing, sequences are first truncated or extended, and then are encoded by PSSM as feature vectors. For a sequence *x* = *R*_1_*R*_2_…*R*_*d*_…, the PSSM representation of *x* is defined as:

$$f_{d}^{PSSM}(x)=(score(R_{1}),score(R_{2}),\ldots ,score(R_{d}))$$

where

$$score(R_{k})=\left\{ \begin{array}{l} m_{k}(R_{k})\;,\;R_{k}\in \{A,C,G,T\}\\ 0\;,\;R_{k}=E \end{array}\right.\;,\;k=1,2,\ldots ,d$$

and *m*_*k*_(*R*_*k*_) represents the score of *R*_*k*_ in the *k*-th column of PSSM, *R*_*k*_ ∈ {*A*,*C*,*G*,*T*}, *k* = 1,2,…,*d*.

#### 2.2.5. Pseudo dinucleotide composition

Pseudo dinucleotide composition (PseDNC) is a feature which considers sequential information as well as physicochemical properties of dinucleotides [23, 24]. PseDNC of sequence *x* is defined as:

$$f_{\lambda ,w}^{PseDnc}(x)=(d_{1}(\lambda ,w,x),\ldots ,d_{16}(\lambda ,w,x),d_{16+1}(\lambda ,w,x),\ldots ,d_{16+\lambda }(\lambda ,w,x))$$

where

$$d_{i}(\lambda ,w,x)=\left\{ \begin{array}{l} \frac{c(\alpha_{i},x)}{\sum_{k=1}^{16}c(\alpha_{k},x)+w\sum_{k=1}^{\lambda }\theta_{k}},\;(1\leq i\leq 16)\\ \frac{w\theta_{i-16}}{\sum_{k=1}^{16}c(\alpha_{k},x)+w\sum_{k=1}^{\lambda }\theta_{k}},\;(17\leq i\leq 16+\lambda ) \end{array}\right.$$

*c*(*α*_*i*_, *x*) denotes the occurrences of dinucleotide *α*_*i*_ in the sequence *x*. The parameter *w* represents the weight factor (default value: 0.05). *λ*, 0 ≤ *λ* ≤ *L* − 2, is the preset integer parameter, denoting the highest counted rank of the correlation along a sequence. *L* represents the length of shortest sequence. *θ*_*k*_ denotes the *k*-rank correlation factor: $\theta_{k}=\frac{1}{L-k-1}\sum_{i=1}^{L-k-1}\frac{1}{n}\sum_{u=1}^{n}{\left(v_{u}(R_{i}R_{i+1})-v_{u}(R_{i+k}R_{i+k+1})\right)}^{2}$, (1 ≤ *k* ≤ *λ*). *R*_*i*_*R*_*i*+1_ represents the *i*-th dinucleotide of sequence *x* and *v*_*u*_(*R*_*i*_*R*_*i*+1_) denotes the value of *u*-th physicochemical indices of *R*_*i*_*R*_*i*+1_. *n* is the number of physicochemical indices. Here, six physicochemical indices: Twist, Tilt, Roll, Shift, Slide and Rise are used [24].

#### 2.2.6. Local structure-sequence triplet elements

Local structure-sequence triplet elements (LSSTE) is an encoding scheme for flexible-length biological sequence [21, 31], which utilizes the piRNA-transposon interaction information.

According to the complementary pairing of the bases: *A* pair with *T*, *C* pair with *G*, there are two statuses: paired and unpaired for each nucleotide in sequences and the relevant transposons. The interaction information is obtained by using RNAplex [12]. Thus, closed brackets: “)” and “(” are used to represent the paired nucleotides of sequences and transposons, respectively, and the dots “.” is used to represent the unpaired nucleotides of both sequences and transposons. For any three adjacent nucleotides (triple) of a sequence, there are 8 possible structural types: “(((”, “((.”, “(.(”, “.((”, “(.”, “.(.”, “.(”, “…”. Further, according to the center nucleotides (*A*, *C*, *G*, *T*) of triples, we can define 32(4×8) different triplet elements: “(((*A*”, “((. *A*”, …,“ …*A*” … “(((*T”*, “((.*T”*, …,“…*T”*. Therefore, the LSSTE feature is defined as the occurrences of these triplet elements in sequences.

### 2.3. Feature Combination-Based piRNA Prediction Models

In the view of information science, a variety of features can bring diverse information, and the combination of various features can lead to better performance than individual features [37–41]. However, the noise between features may adversely influence the feature combination. In order to construct high-accuracy prediction models, we consider two popular feature combination approaches: direct combination and ensemble learning to integrate features. The classifiers are important for building prediction models. Here, we considered several popular classifiers, i.e. random forest (RF) [42], support vector machine (SVM) [43] and logistic regression (LR) [44] etc, and observed that RF can generally produce better performances than other classifiers. Therefore, we finally adopt RF as the basic classifier.

Direct combination is to merge different feature vectors [39]. Ensemble learning uses the weighted average scores of individual feature-based predictors [38, 40]. Given *N* features, we can obtain *N* feature vectors: *v*_1_,*v*_2_,…,*v*_*N*_ for each instance. In the direct combination, we use the merged feature vector *v* = [*v*_1_,*v*_2_,…,*v*_*N*_] to construct prediction models. In the ensemble learning, individual feature-based models are constructed on the training datasets, and the internal cross validation AUC scores of these models are calculated and denoted as *score*_1_, *score*_2_,…,*score*_*N*_. The weights are calculated by

$$w_{i}=\frac{score_{i}}{\sum_{i=1}^{N}score_{i}},\;i=1,2,\ldots ,N.$$

For a testing sequence *x*, *f*_*i*_(*x*) ∈ [0,1] represents the probability of predicting *x* as real piRNAs, *i* = 1,2,…,*N*, and the final predicted results of the ensemble model is given as:

$$F(x)=\sum_{i=1}^{N}w_{i}f_{i}(x)$$

**Fig 1:**
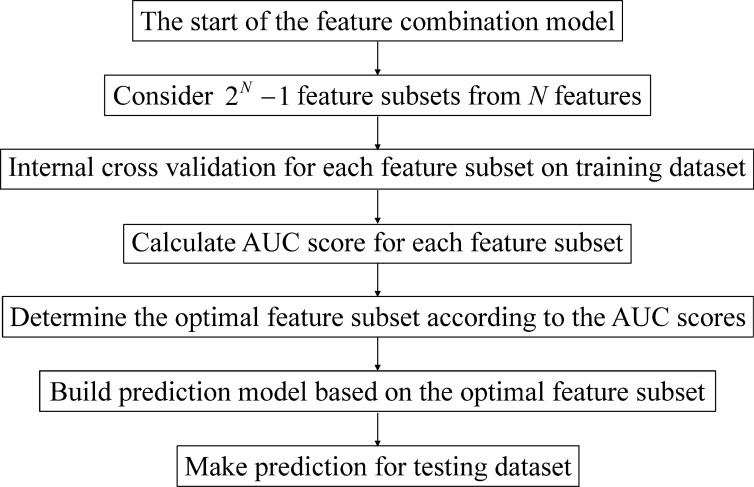
The flowchart of the feature combination model.

In both direct combination and ensemble learning, using all features may not necessarily lead to better performances than using a subset of features. Therefore, which features should be used for feature combination is critical. Here, we develop an approach of determining optimal feature subset and building the feature combination-based prediction models. Given *N* features, there are 2^*N*^ − 1 feature subsets. For each subset, we use the features in the subset and build the prediction model (direct combination or ensemble learning), and the internal cross validation performances of the model on the training set is taken as the evaluation score of the subset. Therefore, the optimal subset with the best AUC score is determined, and prediction model is constructed on the selected features and then is applied to the testing dataset. The flowchart of the feature combination model (direct combination or ensemble learning) is shown in Fig 1.

## 3. Results and Discussion

### 3.1. Performance Evaluation Metrics

The proposed methods are evaluated by the 10-fold cross validation (10-CV). In the 10-CV, a dataset is randomly split into 10 subsets with equal size. For each round of 10-CV, one subset is used as the testing dataset and the rest is considered as the training dataset. The prediction model is constructed on the training dataset, and then it is adopted to predict the testing dataset. This processing is repeated until all subsets are ever used for testing.

Here, we adopt several metrics to assess the performances of prediction models, including the accuracy (ACC), sensitivity (SN), specificity (SP) and the AUC score (the area under the ROC curve). These metrics are defined as:

$$SN=\frac{TP}{TP+FN}$$

$$SP=\frac{TN}{TN+FP}$$

$$ACC=\frac{TP+TN}{TP+TN+FP+FN}$$

where TP, FP, TN and FN are the numbers of true positives, false positives, true negatives and false negatives, respectively. The ROC curve is plotted by using the false positive rate (1-specificity) against the true positive rate (sensitivity) for different cutoff thresholds. Here, we consider the AUC as the primary metric, for it assesses the performance regardless of any threshold.

### 3.2. Evaluation of Various Features

As shown in Table 2, we have six features to develop prediction models. In order to extract diverse information from sequences, we consider different *k*-mers, *k* = 1,2,3,4,5, in spectrum profile, mismatch profile and subsequence profile, and merge feature vectors for each of three profiles. Since mismatch profile, subsequence profile, PSSM and PseDNC have parameters, we discuss how to determine the parameters. Here, random forest (RF) is adopted as the classifier engine, and all prediction models are evaluated on *Human* dataset by using 10-CV.

**Table 2:** Six sequence-derived features.

*Feature*	*Description*	*Parameter*	*Dimension*
***Spectrum Profile***	$f_{1}^{spe}(x)+f_{2}^{spe}(x)+f_{3}^{spe}(x)+f_{4}^{spe}(x)+f_{5}^{spe}(x)$	***No parameters***	***1364***
***Mismatch Profile***	$f_{1,m}^{mis}(x)+f_{2,m}^{mis}(x)+f_{3,m}^{mis}(x)+f_{4,m}^{mis}(x)+f_{5,m}^{mis}(x)$	***m*: *the max mismatches***	***1364***
***Subsequence Profile***	$f_{1,\delta }^{sub}(x)+f_{2,\delta }^{sub}(x)+f_{3,\delta }^{sub}(x)+f_{4,\delta }^{sub}(x)+f_{5,\delta }^{sub}(x)$	*δ****: penalty for the non-contiguous matching***	***1364***
***PSSM***	$f_{d}^{PSSM}(x)$	***d*: *the fixed length of sequences***	*d*
***PseDNC***	$f_{\lambda ,w}^{PseDnc}(x)$	*λ****: the highest counted rank of the correlation along a sequence; w*: *the weight (default value*: *0*.*05)***	16 + *λ*
***LSSTE***	***32 triplet elements***	***No parameters***	***32***

In the mismatch profile, the parameter *m* represents the max mismatches. Here, we assume that *m* does not exceed one third of length of *k*-mers, and obtain $f_{1,0}^{mis}(x)$, $f_{2,0}^{mis}(x)$, $f_{3,1}^{mis}(x)$, $f_{4,1}^{mis}(x)$ and $f_{5,1}^{mis}(x)$, and then merge these vectors to generate the mismatch profile.

**Fig 2:**
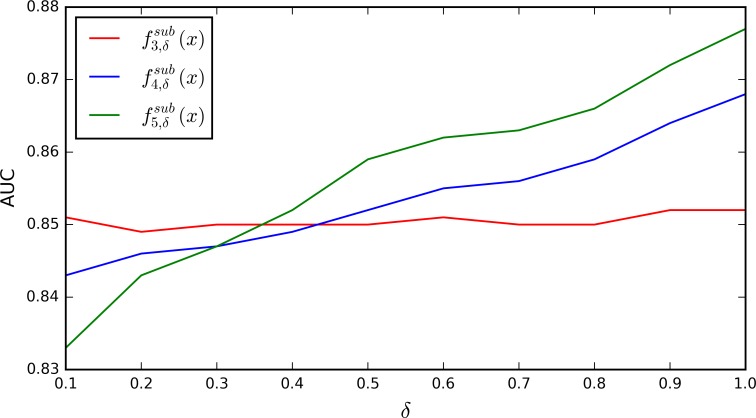
AUC scores of the $f_{k,\delta }^{sub}(x)$ models with the variation of *δ* on *Human* dataset.

In the subsequence profile, the parameter *δ* represents the gap penalty of non-contiguous *k*-mers. Since 1-mer has no gaps and 2-mer includes two nucleotides, we set *δ* as 0 for $f_{1,\delta }^{sub}(x)$ and $f_{2,\delta }^{sub}(x)$. As shown in Fig 2, *δ* = 1 produces the best AUC scores for $f_{3,\delta }^{sub}(x)$, $f_{4,\delta }^{sub}(x)$ and $f_{5,\delta }^{sub}(x)$. Therefore, we merge $f_{1,0}^{sub}(x)$, $f_{2,0}^{sub}(x)$, $f_{3,1}^{sub}(x)$, $f_{4,1}^{sub}(x)$ and $f_{5,1}^{sub}(x)$, and use them as the subsequence profile.

**Fig 3:**
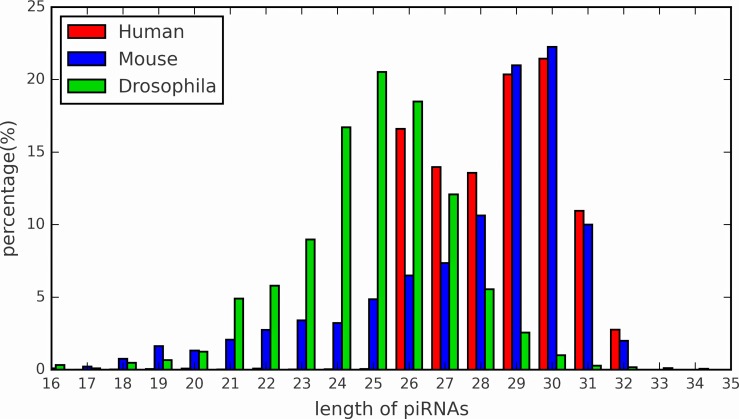
The length distribution of piRNAs in three species.

In the PSSM feature, the parameter *d* represents the fixed length of sequences. Since different species have different length distribution of piRNA sequences. Here, we count the length distribution of piRNAs in three species: *Human*, *Mouse* and *Drosophila*. As show in Fig 3, the length distribution of *Human* and *Mouse* piRNAs reach the peak at 30, whereas that the peak in *Drosophila* is 25. Therefore, we set the parameter *d* as 30. PSSM is calculated on training sequences, and then is used to represent sequences in both training and testing.

**Fig 4:**
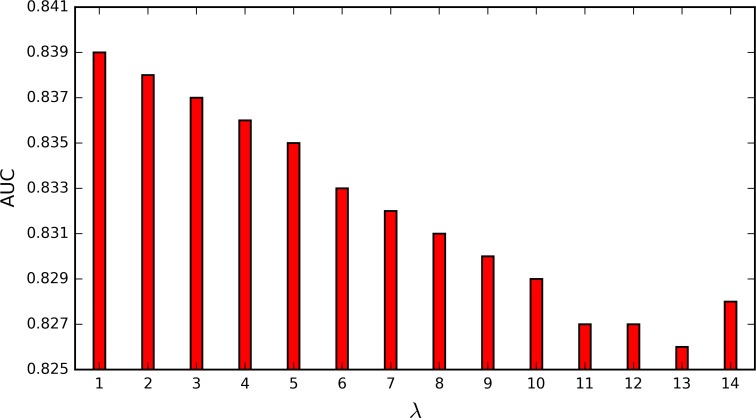
AUC scores of the PseDNC models with the variation of *λ* on *Human* dataset.

The parameter *λ* in PseDNC denotes the additional length of the feature (1 ≤ *λ* ≤ *L* − 2). *L* is the length of shortest piRNA sequences, and is 16 according to Fig 3. To test the impact of parameter *λ*, we construct the prediction models by using different values. As show in Fig 4, the highest AUC score is 0.839 when *λ* = 1. The best parameter is adopted for the following study.

After determining feature parameters, we can compare the capabilities of various features for the piRNA prediction. Here, six features are evaluated on *Human* dataset and the performances of individual feature-based models are obtained by using 10-CV. As shown in Table 3, AUC scores range from 0.695 to 0.881, and the PSSM feature performs best among these features. According to the descending order of AUC scores, features are listed as PSSM, subsequence profile, mismatch profile, spectrum profile, PseDNC and LSSTE. Since performances of LSSTE is much poorer than that of other features, we remove LSSTE and consider five features: spectrum profile, mismatch profile, subsequence profile, PseDNC, PSSM for the final prediction models.

**Table 3:** The performances of six individual feature-based models on *Human* dataset.

*Feature*	*AUC*	*ACC*	*SN*	*SP*
***Spectrum Profile***	***0*.*861***	***0*.*768***	***0*.*778***	***0*.*758***
***Mismatch Profile***	***0*.*866***	***0*.*774***	***0*.*805***	***0*.*742***
***Subsequence Profile***	***0*.*876***	***0*.*793***	***0*.*829***	***0*.*756***
***PSSM***	***0*.*881***	***0*.*808***	***0*.*817***	***0*.*799***
***PseDNC***	***0*.*839***	***0*.*761***	***0*.*778***	***0*.*744***
***LSSTE***	***0*.*691***	***0*.*632***	***0*.*665***	***0*.*599***

### 3.3. Evaluation of Feature Combination-Based Models

Two approaches: direct combination and ensemble learning are adopted to integrate five features and construct high-accuracy prediction models. To avoid the bias of split data, we adopt 10-CV to evaluate the performances of two models.

In each fold of 10-CV, there are 31 (2^5^−1) feature subsets for both direct combination and ensemble learning. The optimal subsets are determined, and are used to build prediction models. As show in Table 4, the direct combination model achieves AUC of 0.917, accuracy of 0.834, sensitivity of 0.857 and specificity of 0.811 on *Human* dataset, and the ensemble learning model achieves AUC of 0.922, accuracy of 0.808, sensitivity of 0.817 and specificity of 0.799. Compared with the individual features-based models, two feature combination models improve AUC of >4%. Clearly, the two models produce much better results, indicating the feature combination approach can effectively combine various features to enhance performances.

**Table 4:** The performances of two feature combination models on three datasets.

*Dataset*	*Method*	*AUC*	*ACC*	*SN*	*SP*
***Human***	***Direct Combination***	***0*.*917***	***0*.*834***	***0*.*857***	***0*.*811***
	***Ensemble Learning***	***0*.*922***	***0*.*808***	***0*.*817***	***0*.*799***
***Mouse***	***Direct Combination***	***0*.*922***	***0*.*844***	***0*.*849***	***0*.*838***
	***Ensemble Learning***	***0*.*926***	***0*.*811***	***0*.*865***	***0*.*758***
***Drosophila***	***Direct Combination***	***0*.*992***	***0*.*957***	***0*.*945***	***0*.*969***
	***Ensemble Learning***	***0*.*994***	***0*.*958***	***0*.*952***	***0*.*965***

In the same way, the direct combination model and ensemble learning model achieve AUC of 0.922 and 0.926 on *Mouse* dataset, respectively. Compared with the piRNA prediction on mammalian: *Human* and *Mouse*, the prediction on *Drosophila* is much better, achieving AUC of 0.992 and 0.994 for the two models. The results on three datasets demonstrate our methods have not only high accuracy but also strong robustness.

Further, we investigate the optimal feature subsets in each fold of 10-CV on three datasets. Statistics is shown in Table 5. We take the results on *Mouse* dataset for analysis. For the direct combination model, the optimal feature subset always consists of spectrum profile and PSSM. For the ensemble learning model, there are two unique optimal feature subsets in ten folds, the subset of spectrum profile and PSSM is determined in nine folds, and the subset of spectrum profile, mismatch profile and PSSM is used once. Several conclusions can be drawn from the statistical results on three datasets. Firstly, the optimal feature subset does not necessarily consist of the highly ranked features, such as the subset of PSSM and subsequence profile. Secondly, the optimal feature subset for the direct combination model or ensemble learning model depends on the training dataset, and determining the optimal subset is necessary for building high-accuracy models.

**Table 5:** The statistical results of the optimal feature subsets in 10-CV on three datasets.

*Dataset*	*Direct Combination*	*Ensemble Learning*
***Human***	***Spectrum+PSSM*:*10***	***Spectrum+PSSM*:*10***
***Mouse***	***Spectrum+PSSM*:*10***	***Spectrum+PSSM*: *9; Spectrum+Mismatch+PSSM*: *1***
***Drosophila***	***Spectrum+PSSM+PseDNC*:*10***	***Spectrum+PSSM*: *1; Spectrum+PSSM+PseDNC*: *9***

### 3.4. Comparison with Other Methods

Here, we adopt two methods: piRNApredictor [13] and Piano [14] as the benchmark methods, and compare our methods with them on three datasets (*Human*, *Mouse* and *Drosophila*). piRNApredictor used the *k*-mer feature (named “spectrum profile” in this paper), and Piano used LSSTE feature. Both methods adopted support vector machine (SVM) to construct prediction models. We implement piRNApredictor to obtain the results. Since the source codes of Piano are available at http://ento.njau.edu.cn/Piano.html, we can run the program on the benchmark datasets. All methods are evaluated on three benchmark datasets by using 10-CV.

As show in Table 6, piRNApredictor and Piano achieve AUC of 0.898 and 0.596 on *Human* dataset, respectively. Our direct combination and ensemble learning produce AUC of 0.917 and 0.922 on the dataset. The proposed methods also yield much better performances than piRNApredictor and Piano on *Mouse* and *Drosophila* datasets. There are several reasons for the superior performances of our methods. Firstly, various useful features can guarantee the diversity for direct combination model and ensemble learning model. Secondly, the direct combination model and ensemble learning model automatically determine the optimal feature subsets on training dataset, for the purpose of incorporating the useful information and avoiding the feature redundancy.

**Table 6:** Comparison between our methods and the state-of-the-art methods.

*Dataset*	*Method*	*AUC*	*ACC*	*SN*	*SP*
***Human***	***Piano***	***0*.*596***	***0*.*564***	***0*.*845***	***0*.*282***
	***piRNApredictor***	***0*.*898***	***0*.*817***	***0*.*861***	***0*.*773***
	***Our Direct Combination***	***0*.*917***	***0*.*834***	***0*.*857***	***0*.*811***
	***Our Ensemble Learning***	***0*.*922***	***0*.*808***	***0*.*817***	***0*.*799***
***Mouse***	***Piano***	***0*.*442***	***0*.*543***	***0*.*842***	***0*.*243***
	***piRNApredictor***	***0*.*893***	***0*.*819***	***0*.*864***	***0*.*774***
	***Our Direct Combination***	***0*.*922***	***0*.*844***	***0*.*849***	***0*.*838***
	***Our Ensemble Learning***	***0*.*926***	***0*.*811***	***0*.*865***	***0*.*758***
***Drosophila***	***Piano***	***0*.*745***	***0*.*694***	***0*.*835***	***0*.*554***
	***piRNApredictor***	***0*.*983***	***0*.*952***	***0*.*927***	***0*.*976***
	***Our Direct Combination***	***0*.*992***	***0*.*957***	***0*.*945***	***0*.*969***
	***Our Ensemble Learning***	***0*.*994***	***0*.*958***	***0*.*952***	***0*.*965***

## 4. Conclusions

The piRNA prediction is an important topic. In this paper, we extract six sequence-derived features to represent piRNA sequences, and integrate these features to develop piRNA prediction models. Compared with other state-of-the-art methods on three datasets, the proposed models have high performances as well as good robustness, which demonstrate that they are promising for transposon-derived piRNA prediction. Here, weights of ensemble learning are determined by the AUC scores of the base predictors, and this strategy is reasonable but arbitrary. We will consider the better way of determining weights for the ensemble learning in the future work. The source codes and datasets are available in supporting information file (S1 File).

## Supporting Information

S1 File The source codes and datasets for piRNA prediction. (ZIP)
