# Supplementary material for: Accurate Prediction of Transposon-Derived piRNAs by Integrating Various Sequential and Physicochemical Features
Source: PLoS One. 2016 Apr 13;11(4):e0153268. doi: 10.1371/journal.pone.0153268 (PMC4830532; doi:10.1371/journal.pone.0153268)
Supplement: S1 File — (ZIP) [file pone.0153268.s001.zip › S1/Manual for transposon-derived piRNA prediction.doc]

**Manual for transposon-derived piRNA prediction**

1. **INTRODUCTION**

The program is developed for piRNA prediction, including six individual feature-based models: spectrum profile, mismatch profile, subsequence profile, PSSM, PseDNC and LSSTE, and two feature combination models: direct combination and ensemble learning. Before the prediction, please make sure your computer has installed the software: RNAplex and the python package: repDNA.

RNAplex (<http://www.bioinf.uni-leipzig.de/~htafer/RNAplex/RNAplex.html>);

repDNA (<http://bioinformatics.hitsz.edu.cn/repDNA/>).

2.**DATASETS**

Here, there are three datasets, covering three species: *Human*, *Mouse* and *Drosophila*. And each dataset includes same number of positive instances and negative instances. Three datasets are included in “S1/Datasets/” with the format of “.fasta”, respectively. For the LSSTE-based model, you need to additional datasets reflecting the piRNA-transposon interaction information, and they are included in “S1/LssteExperiment/AdditionalDatasets/”.

3.**CODES**

All the codes for piRNA prediction are included in “S1/Codes/”. Before the prediction please copy the datasets of the relevant species to the file “S1/Codes/”, and rename the files as “posi_samples.fasta” and “nega_samples.fasta”. Particularly, for the LSSTE-based model, you need to copy the relevant datasets to “S1/Codes/LssteExperiment/”. All predicted results are saved in the file of “.csv”. The shell commands are shown in following.

**Six individual feature-based models**

>>> python SpectrumProfileExperiment.py

>>> python MismatchProfileExperiment.py

>>> python SubsequenceProfileExperiment.py

>>> python PssmExperiment.py

>>> python PsedncExperiment.py

>>> ./LssteExperiment.sh

**Two feature combination models**

>>> python DirectCombinationExperiment.py

>>> python EnsembleLearningExperiment.py
